# Transvaginal ultrasound reliably detects and classifies most cases of deep endometriosis

**DOI:** 10.52054/FVVO.16.2.019

**Published:** 2024-06-28

**Authors:** A Forman

**Affiliations:** Department of Obstetrics and Gynecology, Aarhus University Hospital

This issue of Facts, Views and Vision contains an important consensus statement on imaging-based diagnosis and classification of pelvic deep endometriosis (Condous et al., 2024). This is potentially the most severe form of a chronic gynaecological disease that causes pain and infertility, affects 10% of fertile women and poses a major burden on both individual patients and health care systems. Deep endometriosis may compromise pelvic organ function and represents a surgical challenge. Laparoscopy is still widely used for diagnosis, staging, and evaluation of the need for referral to specialised centres with possibilities for advanced surgery. However, repeated procedures may cause sensitisation and worsen the pain problems that are characteristic of these patients. Adhesion formation may further compromise the possibilities for successful treatment once the patient is referred to the specialist centre (Horne and Missmer, 2022). Detailed, reliable non-invasive diagnosis and classification represent a superior alternative that allows appropriate referral, balanced preoperative assessment of risks and benefits, and forms the platform for a shared decision process.

Fortunately, imaging of deep endometriosis has undergone significant developments in recent years. Major studies have been published on the diagnostic potential of mainly magnetic resonance imaging (MRI) and transvaginal sonography (TVS), but no consensus has been reached on the proper use of these modalities. Moreover, surgery involves different specialties including colorectal surgery and urology apart from gynaecology and these have their preferred approach to pelvic imaging. Finally, the potential for non- invasive staging according to available systems needs critical analysis, and imaging standards for future research purposes have not been defined.

The consensus statement presented here (Condous et al., 2024) signifies a substantial step forward. As many as eight international scientific societies each with their individual profile and basis collaboratively made an impressive, structured effort to assess the current status of imaging for proper assessment of endometriosis. A systematic approach based on a published protocol was used, with an initial critical review of the literature and preliminary statements based on proper statistical methods. These statements underwent rigorous refinement through an iterative process involving 50 experts with different profiles from leading centres. A final version was approved by all participants. This meticulous process ensured the derivation of valid statements firmly rooted in both scientific evidence and contemporary best practice.

Notably, TVS conducted by skilled expert operators emerged as a potent diagnostic tool albeit with certain constraints. The performance of MRI mirrored that of TVS in most aspects, except for parametrial affection and endometriosis nodules distant to the probe on the sigmoid, where MRI demonstrated superiority. Limited data were available on CT and use of this modality was considered less appropriate due to radiation exposure.

The authors also reviewed the performance of TVS and MRI for non-invasive classification of deep endometriosis according to systems in current clinical use. When applied in combination with the #Enzian system (Keckstein et al., 2021; Hudelist et al., 2021), both techniques reliably described most cases of deep and ovarian endometriosis with some reservations for parametrial lesions, and the expert panel strongly agreed on the use of this approach. TVS for other classification systems was found to be less useful for various reasons including insufficient evidence and had mixed support from the panel.

The prospect of using TVS, a readily available tool in clinical settings, to accurately detect and describe most cases of deep endometriosis according to the increasingly accepted #Enzian classification is highly promising. With positive findings, laparoscopy for diagnosis and staging may be omitted and patients can be referred to expert centres without delay. In cases where clarity is lacking, MRI can serve as a diagnostic alternative. This offers possibilities for revision of current clinical strategies. However, it is crucial to acknowledge that these promising outcomes were achieved by highly skilled operators and the predictive values may not readily translate to general clinical settings. Particularly, the risk of false negative findings necessitates careful consideration, especially in cases with symptoms or clinically abnormal findings. It is therefore imperative that this consensus paper inspires the development of educational programmes with certification aimed at both primary and secondary level clinical practice and further refined expertise in advanced tertiary centres. Clinicians at all levels must acknowledge their individual limitations to mitigate diagnostic errors and consider the influence of prevalence on predictive values, as recently discussed by Koninckx et al. (2023). Referral centres should offer easy access to expert TVS for colleagues in gynaecological practice without the necessary training and experience.

Lastly, although deep and ovarian endometriosis is effectively diagnosed and classified, no imaging technique has so far been able to reliably detect peritoneal disease. Severe cases of this phenotype are probably related to pain, but the pathogenetic process leading to clinical problems has not been definitively established; thus, peritoneal endometriosis is found in approximately 20% of fertile women without pelvic problems (Moen and Muus, 1991).

In future practice, achieving a comprehensive non-invasive classification will be essential for optimising outcomes when surgery is planned. Patients should undergo as few interventions as possible, preferably only one, to minimise the risk of complications and sensitisation with worsened pain induced by the surgical procedure per se. Non-invasive detailed diagnosis should also be the rule when patients are offered medical treatment, which seems feasible for a significant proportion of patients with deep disease (Egekvist et al., 2019). Future research publications should include these data, irrespective of the treatment modality, and non-invasive imaging must be acknowledged as a subspecialty aligned with advanced endometriosis surgery, emphasizing its importance in the overall management approach.
